# Association between sleep-disordered breathing and breast cancer aggressiveness

**DOI:** 10.1371/journal.pone.0207591

**Published:** 2018-11-21

**Authors:** Francisco Campos-Rodriguez, Antonio Cruz-Medina, Maria Jose Selma, Maria Rodriguez-de-la-Borbolla-Artacho, Adrian Sanchez-Vega, Francisco Ripoll-Orts, Carmen V. Almeida-Gonzalez, Miguel Angel Martinez-Garcia

**Affiliations:** 1 Respiratory Department, Hospital Universitario de Valme, Sevilla, Spain; 2 Instituto de Biomedicina de Sevilla (IBIS), Universidad de Sevilla, Sevilla, Spain; 3 CIBERES. Instituto de Salud Carlos III. Madrid, Spain; 4 Respiratory Department, Hospital Universitario y Politécnico La Fé, Valencia, Spain; 5 Oncology Department, Hospital Universitario de Valme, Sevilla, Spain; 6 Breast cancer Unit. Hospital Universitario y Politécnico La Fé, Valencia, Spain; 7 Biostatistics Unit. Hospital Universitario de Valme, Sevilla, Spain; University of South Alabama Mitchell Cancer Institute, UNITED STATES

## Abstract

**Background:**

Sleep-disordered breathing (SDB) has been associated with cancer aggressiveness, but studies focused on specific tumors are lacking. In this pilot study we investigated whether SDB is associated with breast cancer (BC) aggressiveness.

**Methods:**

83 consecutive women <65 years diagnosed with primary BC underwent a home respiratory polygraphy. Markers of SDB severity included the apnea-hypopnea index (AHI) and the 4% oxygen desaturation index (ODI4). The Ki67 proliferation index, lack of hormone receptors (HR-), Nottingham Histological Grade (NHG), and tumor stage were used as markers of BC aggressiveness. The association between SDB and molecular subtypes of BC was also assessed.

**Results:**

The mean (SD) age was 48.8 (8.8) years and body mass index was 27.4 (5.4) Kg/m2. 42 women (50.6%) were post-menopausal. The median (IQR) AHI was 5.1 (2–9.4), and ODI4 was 1.5 (0.5–5.8). The median (IQR) AHI did not differ between the groups with Ki67>28% and Ki67<29% [5.1 (2.6–8.3) vs 5.0 (1.5–10), p = 0.89)], HR- and HR+ [5.7 (1.6–12.4) vs 4.9 (2–9.4), p = 0.68], NHG (Grade3, Grade2, and Grade1; p = 0.86), tumor stage (stage III-IV, stage II, and stage I; p = 0.62), or molecular subtypes (Luminal A, Luminal B, HER2, and triple negative; p = 0.90). The prevalence of an AHI≥5 did not differ between the groups with Ki67>28% and Ki67<29% (51.2% vs 52.3%, p = 0.90), HR- and HR+ (58.3% vs 49.1%, p = 0.47), NHG categories (p = 0.89), different tumor stages (p = 0.71), or molecular subtypes (p = 0.73). These results did not change when the ODI4 was used instead of the AHI.

**Conclusion:**

Our results do not support an association between the presence or severity of SDB and BC aggressiveness.

## Introduction

Sleep-disordered breathing (SDB) is a common disorder affecting about 13% of men and 6% of women of the general population and is characterized by repetitive obstruction of the upper airway that provokes intermittent hypoxia (IH) and sleep fragmentation.[1,2] These two consequences of SDB have been associated with excessive daytime sleepiness (EDS), impaired quality of life, increased risk of traffic accidents, hypertension and other cardiovascular disorders.[3] Recently, SDB has also been associated with increased cancer incidence and mortality, and IH has been identified as the possible common pathway between both disorders.[4–6] However, most of this evidence rests on studies with methodological flaws, since they were either retrospective, or not originally designed to assess this association, or they did not analyze specific types of tumor.[7]

Breast cancer (BC) is the most common cancer and the second leading cause of cancer death in women.[8] Basic research has reported that intra-tumoral hypoxia is associated with an increased risk of BC metastasis and greater patient mortality and that these outcomes are more associated with IH than with continuous hypoxia.[9,10] Considering that IH is one of the hallmarks of SDB,[11] it is reasonable to hypothesize an association between SDB and BC. However, although the effect of sleep duration and night shift work on BC has been extensively analyzed, the relationship with SDB has barely been investigated.[12–17] Two retrospective studies, both using a nationwide database, have found that women with a diagnosis of sleep apnea had a twofold increased risk of developing BC.[18,19] The association between SDB and BC aggressiveness, however, has not yet been addressed. Thus, the aim of this pilot study was to investigate whether the prevalence or severity of SDB is associated with BC aggressiveness.

## Methods

### Design and participants

We conducted a pilot, cross-sectional study. Consecutive women aged 18 to 65 years and diagnosed with primary BC at two Spanish University Hospitals between October 2015 and October 2017 were eligible for this study. Valme Hospital is located in the South of Spain, whereas La Fe Hospital is located in the East. Both of them are tertiary University hospitals and cover rural as well as metropolitan areas. Both of them belong to the Spanish National Public Health System. The diagnosis of BC was always based on a biopsy sample assessed by an experienced pathologist. BC was considered as primary if the woman had no previous history of BC. Women were excluded if they had respiratory or heart failure, a prior diagnosis of SDB, or previous CPAP treatment. The study was approved by the Ethics Committee of both centers (Comité de Ética de la Investigación Sevilla Sur, and CEIC Instituto para la Investigación Sanitaria La Fe) and all the participants provided informed signed consent.

### Study procedures

Women diagnosed with primary BC were recruited at the Oncology or Breast Cancer Unit. Information regarding BC was collected by an oncologist or breast surgeon, based on the histology report. The women were then referred to the Sleep Unit, where they completed a demographic, clinical, and sleep questionnaire and underwent a sleep study the same night. No more visits were programmed in this study.

### Measurements

#### Demographic, clinical, and sleep information

This information was collected in accordance with a questionnaire specifically developed for this study which included general and anthropometric data, menopausal status, history of hormone replacement therapy (HRT) and hereditary BC, subjective sleep duration, and night shift work. ESD was assessed by the validated Spanish version of the Epworth Sleepiness Scale (ESS).[20] All the data were collected in the same day.

#### Breast cancer data

BC data was obtained from the histology report and included information on the molecular subtype,[8,21] tumor cell proliferation measured by the Ki67,[8,22] presence or absence of estrogen and progesterone hormone receptors (HR),[8,21] histologic grading by means of the Nottingham Histological Grade (NHG),[23] and tumor stage according to the TNM staging system.[24] A further description of all these variables can be found in the **S1 Appendix.**

#### Sleep study

Every women underwent a home respiratory polygraphy using a device validated against polysomnography and following the Spanish Society of Pneumology and Thoracic Surgery Guidelines for SDB diagnosis and treatment.[25] Every sleep study was manually scored by skilled staff. All the studies included continuous recording of the oro-nasal flow and pressure, respiratory movements, and oxyhemoglobin saturation (SaO2). Apnea was defined as complete cessation of oro-nasal flow for ≥10 seconds and was classified as either obstructive or central, based on the presence or absence of respiratory efforts. Hypopnea was defined as a 30–90% reduction in oro-nasal flow for ≥10 seconds followed by a ≥3% decrease in SaO2. A sleep study was considered valid if at least 4 hours of recording and more than 3 hours of subjective sleep were reported. Invalid studies were repeated. The apnea-hypopnea index (AHI) was defined as the number of apneas plus hypopnea per hour of recording. The following oximetric variables were also obtained: percent time spent with SaO2<90% (T90), oxygen desaturation index at 3% (ODI3) and 4% (ODI4), and minimum SaO2 (SaO2min). SDB was defined as an AHI≥5, and moderate-to-severe SDB as an AHI≥15.[26]

### Endpoints

The endpoint of this study was the association between the prevalence and severity of SDB and the aggressiveness of BC. The Ki67 value was selected as the main variable indicative of the aggressiveness of the BC, and was categorized into two groups, based on the median value of our sample, which was 28%. Since this value is within the range of 20–29% usually used to define high proliferation in BC,[8,22] our cut-off point adequately separates women with more and less aggressive BC. The molecular subtypes of BC, lack of estrogen and progesterone hormone receptors (HR-), the NHG, and the tumor stage were used as secondary markers of aggressiveness.

### Statistics

The sample size was calculated by assuming that the group with less aggressive BC had an AHI similar to that of the women in the general population.[27] At least 41 women in each group would be necessary to show an increase of at least 5 points in the AHI in the group with more aggressive BC, assuming an SD of 7, a power of 90% and an α error of 0.05.

The IBM SPSS 19.0 statistical package (SPSS Inc. Chicago, IL, USA) was used for data processing and analysis. Results are expressed as mean (SD) or median (interquartile range -IQR-) for continuous variables and number of patients (%) for categorical variables. Normality in the variables distribution was assessed with the Shapiro-Wilk test.

The student t-test or Mann-Whitney test was used to compare the sleep variables between the group with more and less aggressive BC (Ki67>28% vs <29%) and those with HR- vs HR+, and the ANOVA or Kruskal-Wallis test was used to compare the sleep parameters between the molecular subtypes, categories of the NHG and tumor stage, whereas the Chi-square or Fisher exact test was used to compare SDB prevalence between groups.

Correlation analyses (Spearman test) were used to investigate the association between the AHI and ODI4 with the Ki67, NHG and tumor stage as continuous variables.

Two-tailed p-values of <0.05 were considered significant.

## Results

**Fig 1:**
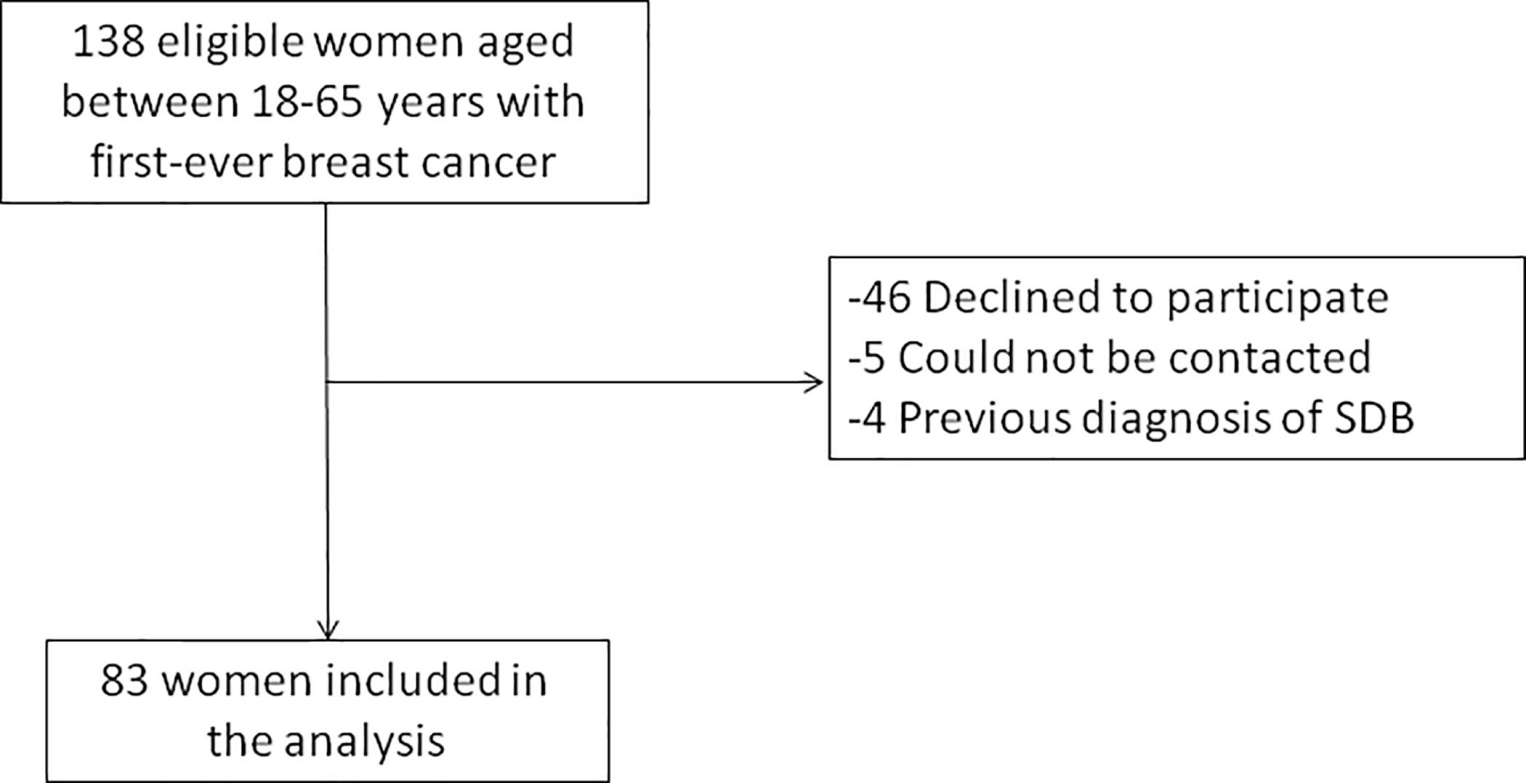
Flow chart of the study.

Of the 138 eligible women, 83 were finally included in the study (**Fig 1**). The median time from BC diagnosis to the date of the sleep study was 68 (IQR 48–87) days. The baseline general characteristics of the women, the results of the sleep studies, and the most salient features of the BC histology are shown in **Table 1.** Except age and anthropometric variables, all other variables showed a non-normal distribution. Women had a mean (SD) age of 48.8 (8.8) years, body mass index (BMI) of 27.4 (5.4) Kg/m2, and 50.6% were postmenopausal. These baseline general characteristics did not differ between the group with more and less aggressive BC **(S1 Table)**. The median (IQR) AHI was 5.1 (2–9.4), the ODI4 was 1.5 (0.5–5.8), and the prevalence of SDB was 51.8%. All cases diagnosed with SDB corresponded to obstructive sleep apnea, and none of the women presented central sleep apnea.

**Table 1:** Baseline characteristics of the women.

Variables	Whole sample(n = 83)
*General characteristics*	
Age (years)	48.8 (8.8)
Body mass index (Kg/m2) Body mass index≥30 Kg/m2	27.4 (5.4) 24 (28.9%)
Neck circumference (cm)	34.4 (3.1)
Waist-to-hip ratio	0.85 (0.09)
Post-menopausal status	42 (50.6%)
Hormone replacement therapy	3 (3.6%)
Familiar history of breast cancer	22 (26.5%)
Active smoking	15 (18.1%)
Night shift work >1 year	15 (18.1%)
Number of hours of sleep in the last year	7 (6–8)
Epworth scale Epworth scale>10	5 (3–8) 10 (12%)
*Sleep Study*	
Sleep study time (min)	414 (360–449)
AHI (events per hour) AHI≥5 AHI≥15	5.1 (2–9.4) 43 (51.8%) 11 (13.3%)
Central AHI (events per hour)	0 (0–0.2)
ODI4 (desaturations per hour)	1.5 (0.5–5.8)
ODI3 (desaturations per hour)	5.2 (2–10.1)
Time spent with oxygen saturation <90%	0 (0–0.70)
Minimum oxygen saturation	89 (85–91)
*Histological characteristics of the breast cancer*	
Histological type	
Infiltrating ductal Others	77 (92.8%) 6 (7.2%)
Molecular subtype	
Luminal A Luminal B HER2+ Triple negative	20 (24.1%) 22 (26.5%) 21 (25.3%) 20 (24.1%)
Ki67 value	28 (15–45)
Nottingham histological grade	
Grade1 Grade2 Grade3	17 (20.5%) 39 (47%) 27 (32.5%)
Absence of estrogen and progesterone hormone receptors	21 (25.3%)
Tumor stage	
Stage I Stage II Stage III+IV	37 (44.6%) 34 (41%) 12 (14.5%)
Histological type	
Infiltrating ductal Others	77 (92.8%) 6 (7.2%)
Molecular subtype	
Luminal A Luminal B HER2+ Triple negative	20 (24.1%) 22 (26.5%) 21 (25.3%) 20 (24.1%)
Ki67 value	28 (15–45)
Nottingham histological grade	
Grade1 Grade2 Grade3	17 (20.5%) 39 (47%) 27 (32.5%)
Absence of estrogen and progesterone hormone receptors	21 (25.3%)
Tumor stage	
Stage I Stage II Stage III+IV	37 (44.6%) 34 (41%) 12 (14.5%)

AHI: apnea-hypopnea index; ODI: oxygen desaturation index.

Data are expressed as mean (SD), median (first-third quartile), or number of patients (%).

### Comparison of markers of SDB severity according to the Ki67 value

**Fig 2:**
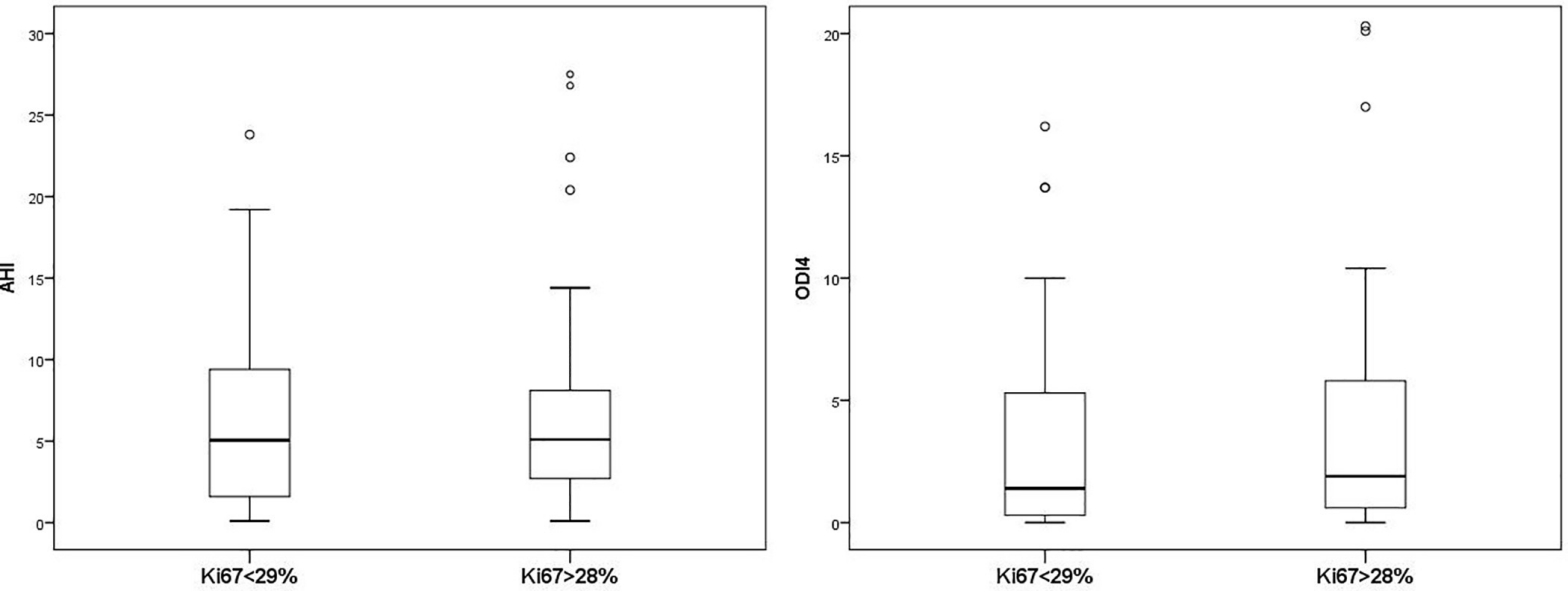
Comparison of markers of SDB severity in the groups of breast cancer with greater and lesser cell proliferation, as assessed by the Ki67. SDB = sleep-disordered breathing. AHI = apnea-hypopnea index. ODI4 = oxygen desaturation index at 4%. The Mann-Whitney test was used for comparisons.Left panel, AHI values: Ki67<29%, median 5.0 (IQR 1.5–10). Ki67>28%, median 5.1 (IQR 2.6–8.3), p = 0.89.Right panel, ODI4 values: Ki67<29%, median 1.4 (IQR 0.3–5.4). Ki67>28%, median 1.9 (IQR 0.5–5.8), p = 0.57.

Compared to women with less aggressive BC, as assessed by a Ki67<29%, those with more aggressive BC (Ki67>28%) did not show differences in the AHI (median 5.0, IQR 1.5–10, vs 5.1, IQR 2.6–8.3, p = 0.89), or ODI4 (median 1.4, IQR 0.3–5.4 vs 1.9, IQR 0.5–5.8, p = 0.57) (**Fig 2**). Similarly, no differences were found in the other oximetric parameters between groups (**S2 Table**).

When the Ki67 was analyzed as a continuous variable, no correlations were observed between the Ki67 and the AHI (r = 0.03, p = 0.74) or the ODI4 (r = 0.09, p = 0.37).

### Comparison of markers of SDB severity according to secondary markers of BC aggressiveness

**Fig 3:**
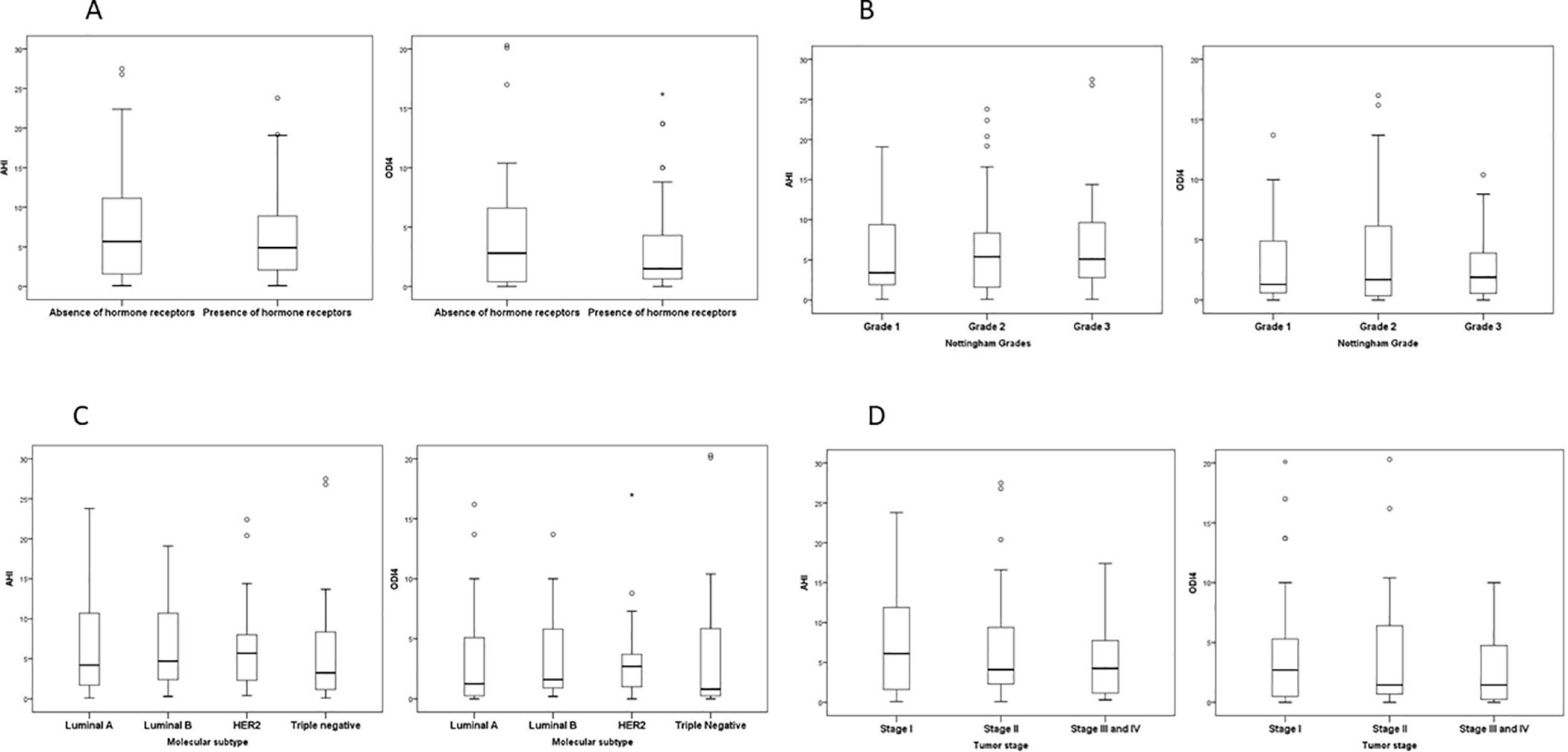
Comparison of markers of SDB severity according to the secondary markers of breast cancer aggressiveness. SDB = sleep-disordered breathing. AHI = apnea-hypopnea index. ODI4 = oxygen desaturation index at 4%. *Panel A, presence of estrogen and/or progesterone hormone receptors vs absence of estrogen and progesterone hormone receptors. The Mann-Whitney test was used for comparisons. AHI values: absence of hormone receptors, median 5.7 (IQR 1.6–12.4; presence of hormone receptors, median 4.9 (IQR 2–9.4), p = 0.68ODI4 values: absence of hormone receptors, median 2.8 (IQR 0.3–6.9), presence of hormone receptors, median 1.5 (IQR 0.6–4.9), p = 0.65 *Panel B, Nottingham Histological Grade. The Kruskal-Wallis test was used for comparisons. AHI values: Grade 1, median 3.4 (IQR 1.7–9.4), Grade 2, median 5.4 (IQR 1.2–8.4), Grade 3, median 5.1 (IQR 2.5–10.7); p = 0.86. ODI4 values: Grade 1, median 1.3 (IQR 0.4–5.1), Grade 2, median 1.7 (IQR 0.3–6.4), Grade 3, median 1.9 (IQR 0.5–4.3); p = 0.90. *Panel C, molecular subtypes of the breast cancer. The Kruskal-Wallis test was used for comparisons. AHI values: Luminal A, median 4.2 (IQR 1.6–11.3), Luminal B, median 4.7 (IQR 2.3–11.0), HER2, median 5.7 (IQR 2.3–8.1), triple negative, median 3.2 (IQR 0.9–8.4); p = 0.90. ODI4 values: Luminal A, median 1.2 (IQR 0.2–5.2), Luminal B, median 1.6 (0.8–6.1), HER2, median 2.7 (IQR 0.8–5.0), triple negative, median 0.8 (IQR 0.2–5.8); p = 0.71. *Panel D, tumor stage. The Kruskal-Wallis test was used for comparisons. AHI values: Stage I, median 6.1 (IQR 1.6–11.9), Stage II, median 4.1 (IQR 2.3–9.7), Stage III and IV, median 4.2 (IQR 0.7–7.9); p = 0.62. ODI4 values: Stage I, median 2.7 (IQR 0.4–5.5), Stage II, median 1.4 (IQR 0.6–6.6), Stage III and IV, median 1.4 (IQR 0.2–5.2); p = 0.77.

The AHI and ODI4 values did not vary among the different molecular subtypes, categories of the NHG and tumor stages, or presence or absence of HR (**Fig 3**).

These results did not change when other oximetric parameters were analyzed (**S2 Table**).

When the categories of the NHG and tumor stage were analyzed as continuous variables, no correlations were observed between the NHG grades and AHI (r = 0.05, p = 0.62) or ODI4 (r = 0.04, p = 0.66), or between the tumor stage categories and AHI (r = -0.09, p = 0.37) or ODI4 (r = -0.05, p = 0.64).

### Prevalence of SDB according to the categories of different markers of BC aggressiveness

**Fig 4:**
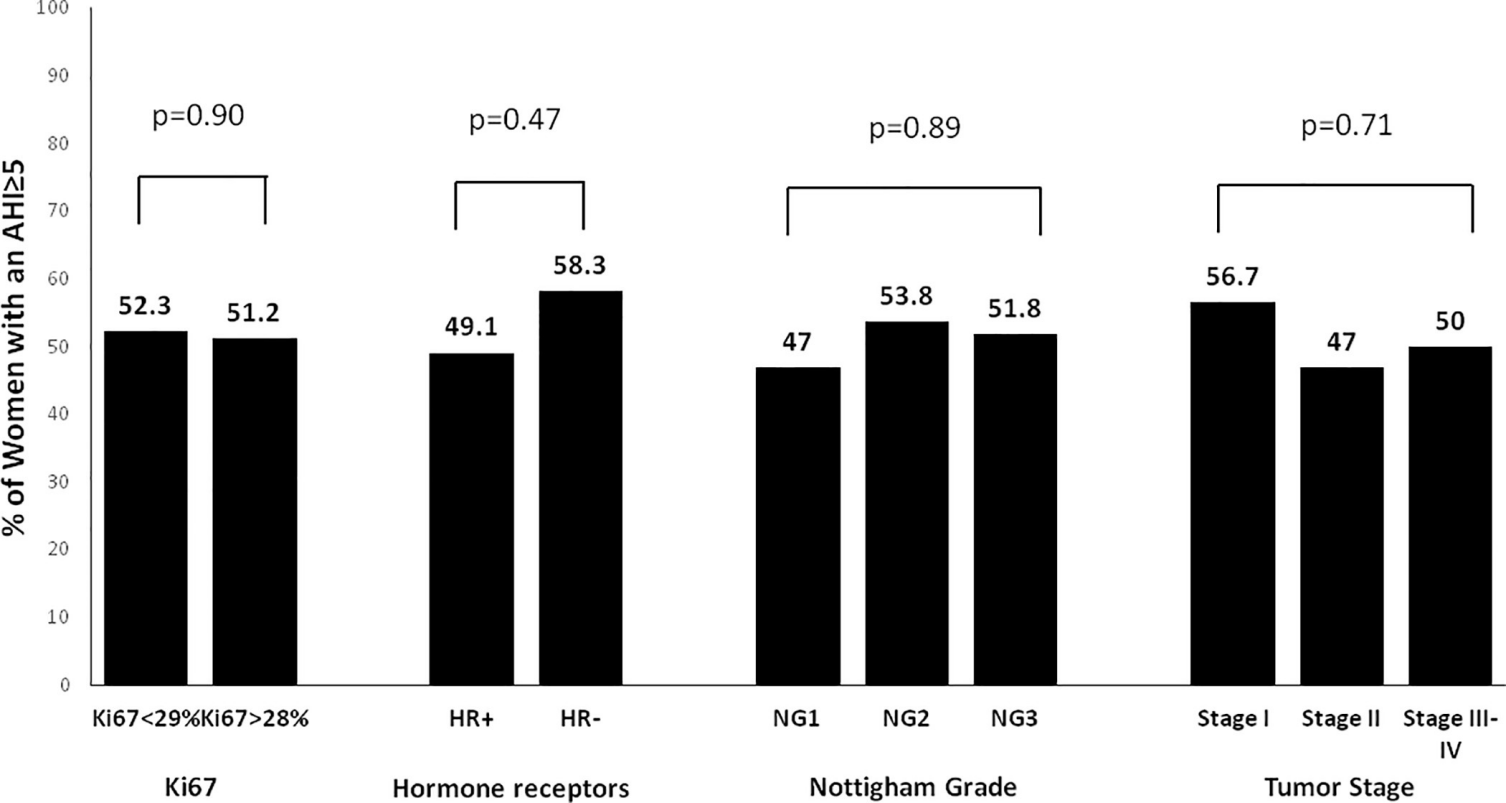
Prevalence of SDB in the categories of the different markers of breast cancer aggressiveness. SDB = sleep-disordered breathing. AHI = apnea-hypopnea index.

**Fig 5:**
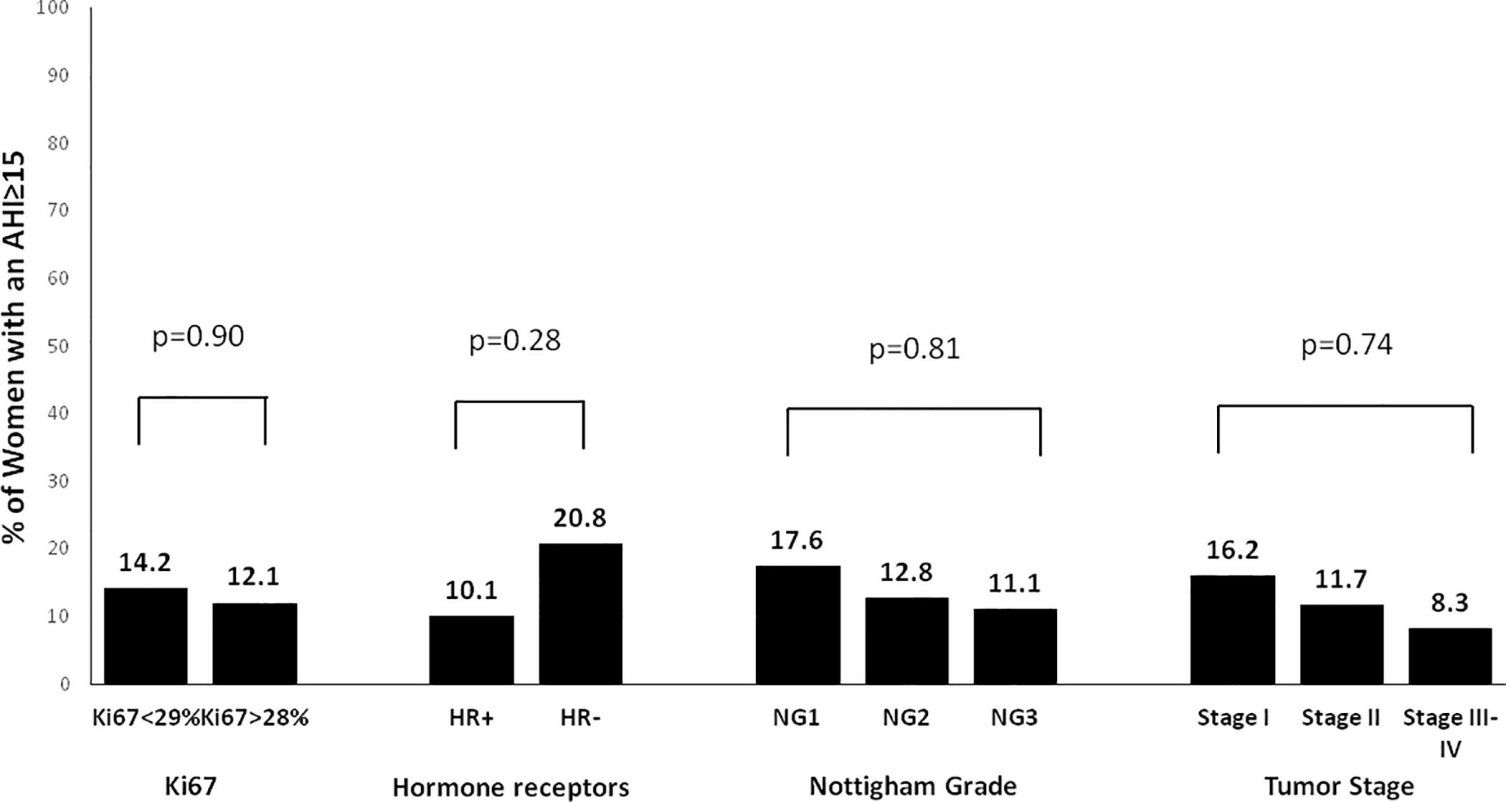
Prevalence of moderate-to-severe SDB in the categories of the different markers of breast cancer aggressiveness. SDB = sleep-disordered breathing. AHI = apnea-hypopnea index.

**Fig 6:**
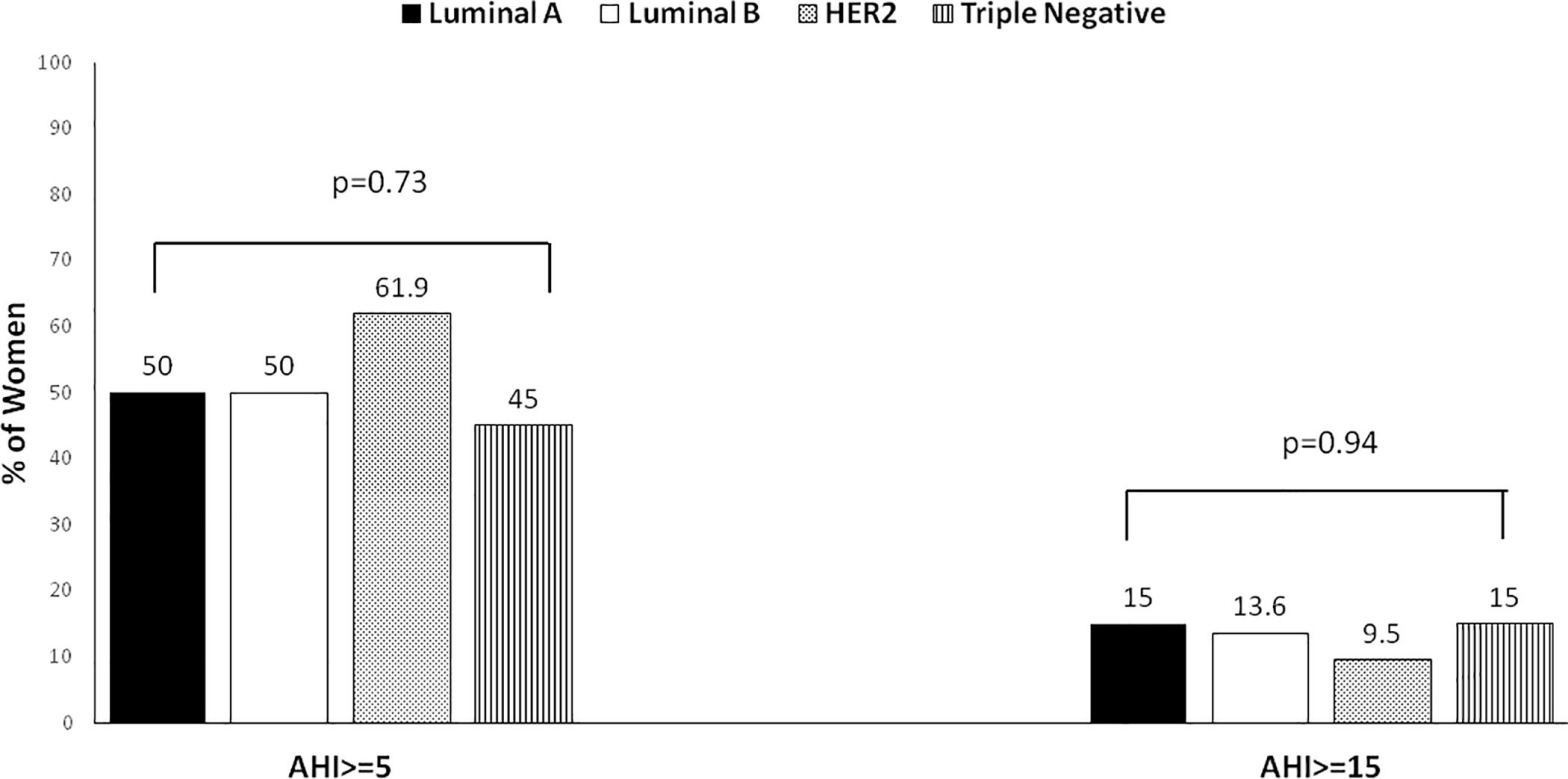
Prevalence of SDB, and moderate-to-severe SDB in the categories of the different molecular subtypes of breast cancer. SDB = sleep-disordered breathing. AHI = apnea-hypopnea index.

According to the Ki67, women with more and less aggressive BC had a similar prevalence of SDB (51.2% vs 52.3%, p = 0.90) and moderate-to-severe SDB (12.1% vs 14.2%, p = 0.90) (**Figs 4 and 5**). The prevalence of SDB and moderate-to-severe SDB was similar between the different molecular subtypes of BC (**Fig 6**). Likewise, there were no differences in the prevalence of SDB and moderate-to-severe SDB between the categories of the rest of secondary markers of BC aggressiveness (**Figs 4 and 5**).

None of these previous results varied when the women were separately analyzed according to the menopausal status.

## Discussion

The results of this pilot study, specifically designed to address SDB in middle-aged women with primary BC, show that there is no association between the AHI and a wide range of oximetric parameters, including direct measures of IH, and several markers of BC aggressiveness. Likewise, we did not find any association between SDB and any particular molecular subtype of BC. These results suggest a lack of association between SDB and BC aggressiveness.

In recent years, there has been great interest in the association between SDB and cancer. IH mimicking SDB has been shown to enhance tumor growth and increase metastatic spreading in animal models.[6,28] Furthermore, human studies have reported a relationship between SDB severity and greater incidence, aggressiveness and poorer prognosis of cancer.[4,5,29] However, these human studies were limited by their retrospective design or the fact that these cohorts were not originally designed to address this association. Another flaw was that most of them analyzed all types of cancer.[7] However, the impact of SDB may differ from one type of tumor to the other, since different types of malignant cells may have different adaptive responses to IH, so there is a need to investigate the association between SDB and specific cancer sites.[7,30–32] However, since basic research in animal models has been restricted to a few types of cancer cells (mostly melanoma cells), it is not known which cancers are more susceptible to being affected by IH, which precludes conducting large studies on this topic.

In women, BC is the leading cause of cancer, and the second most common cause of cancer death.[8] Some non-breathing sleep disorders such as circadian disruption due to night-shift work and short sleep duration have been associated with greater BC incidence and morbidity, particularly the former,[12–17] and two studies using a national insurance longitudinal database observed an increased incidence of BC in women diagnosed with SDB, compared to those without SDB.[18,19] Evidence about the effect of SDB and IH on the aggressiveness of this tumor is lacking, however, even though it is reasonable to hypothesize that SDB may influence BC aggressiveness. This hypothesis is further supported by basic studies showing that intra-tumoral hypoxia and exposure of BC cells to hypoxia is associated with a significantly increased risk of metastasis and patient mortality, usually mediated by hypoxia-inducible factors (HIFs) that promote tumor vascularization and growth.[33,34] Furthermore, recent studies have found that IH confers pro-metastatic gene expression on BC cells,[35] and that this effect is more pronounced with IH than with continuous hypoxia.[9,10] Considering that IH is one of the hallmarks of SDB, and that increased HIF transcription has also been reported in SDB, a pathophysiological link between SDB and BC seems plausible.[11,30,36]

Our results do not support this hypothesis, however, since both SDB prevalence and markers of SDB severity, such as the AHI, the ODI4 (a direct marker of IH), and other oximetric parameters, were similar in women with more and less aggressive BC. It should be noted that in our study BC aggressiveness was not based on a single variable as we collected a wide range of histological markers of poorer BC prognosis such as the Ki67 (a surrogate of cell proliferation), the absence of HR, the NHG (which assesses tumor differentiation), and the tumor stage.[8,22,23] BC is currently classified into molecular subtypes which have different biological properties and prognosis; the HER2 and triple negative subtypes are recognized as being the most aggressive and as provoking the poorest prognosis.[21] Even though these molecular subtypes are based on the aforementioned markers of aggressiveness, we also analyzed the four subtypes of BC but we found that none of them was associated with any marker of SDB severity or prevalence. Thus, our findings suggest a true lack of effect of SDB and IH on this type of cancer.

One possible explanation for our findings is that, although the prevalence of SDB among women with BC was as high as 51.8%, most of them had a very mild form of the disease, with a median AHI of 5.1/h. Furthermore, the differences in the AHI between the categories of the different markers of aggressiveness used in this study were very slight, usually between <1 and 2 events/hour. This suggests that even in the case of a much larger sample size, or a statistically significant difference, most researchers would agree that such a small variation in AHI of only 1–2 points between groups is clinically irrelevant and would not support a role for SDB in BC aggressiveness. This mild SDB severity was also reflected by a low ODI4 of only 1.5 desaturations/hour, which is likely to have a very limited effect on BC. Thus, although it cannot be ruled out that BC cells may be resistant to IH or other mechanisms triggered by SDB and, in fact, there are studies in mouse models showing that oxidative stress induced by hypoxia/reoxygenation is not a major factor driving progression of already established BC tumors,[37] we believe that the most likely explanation for the negative results in our series is that SDB did not have enough severity to influence BC aggressiveness.

The results of this study can only apply to women aged up to 65 years old. Our decision to exclude older women was based on previous studies showing that the association between SDB and cancer seems to be restricted to middle-age patients. In a retrospective Spanish multicenter study with nearly 5,000 patients, the association between SDB and cancer incidence and mortality was reported only in subjects <65 years while it disappeared in those aged over 65 years.[4,29] In agreement with these data, recent basic research has shown that IH mimicking SDB increases tumor progression in young female mice but has no effect on tumor growth in aged females; this finding was attributable to changes in the recruitment and function of tumor-associated macrophages and impaired immune response in aged female mice.[38] Based on this evidence, we chose to exclude older women to avoid any potential bias by selecting women with lower cancer risk associated with SDB.

Our study has limitations. First, it was designed as a pilot study and, accordingly, the analyzed sample is small. However, the sample size was calculated to detect differences in the primary endpoint, various markers of SDB severity and BC aggressiveness were used, and different molecular subtypes of BC were also analyzed, so we believe it unlikely that the results would have changed with a larger sample size. Second, women were diagnosed by means of home respiratory polygraphy, instead of a conventional polysomnography. This may have underestimated the prevalence and severity of SDB.[39] However, this limitation would have equally applied to women with more and less aggressive BC, so we do not think that it had any influence on the lack of association observed. Third, we excluded women aged over 65 years, so we cannot rule out a possible association between SDB and BC in this population. The reason for this decision has been explained in detail above. Furthermore, recent data have shown that menopause is a more important determinant of SDB prevalence than age in middle-aged females, and half of our series comprised postmenopausal women.[2] Finally, some variables such as the intensity and duration of the light the women were exposed to at night, light-dark cycles, diet, and environmental conditions—all of which may affect sleep and the duration of REM and non-REM sleep stages, as well as being related to BC- were not assessed in this study. However, since no association was found between SDB and BC, the possible confusing effect of these factors should have been scarce.

In conclusion, our study conducted in middle-aged women with primary BC does not support an association between the presence or severity of SDB and the aggressiveness of BC measured by various markers, or an association between SDB and any particular molecular subtype of BC. Given that research on SDB and cancer is currently focused on identifying specific cancer sites that may be susceptible to the consequences of SDB such as IH and sleep fragmentation, the results of our pilot study on BC are important because they discourage the organization of any larger and costlier studies on this type of cancer.

## Supporting information

S1 Appendix Methods. (DOCX)

S1 Table Comparison of baseline general characteristics between the groups of women with more (Ki67>28%) and less aggressive (Ki67<29%) breast cancer. p-value was >0.05 for all comparisons between the group with Ki67>28% and Ki67<29%
Data are expressed as mean (SD), median (first-third quartile), or number of patients (%).
(DOCX)

S2 Table Values for different oximetric parameters of SDB according to the categories of several aggressiveness markers of breast cancer. SDB: sleep-disordered breathing. HR-: absence of estrogen and progresterone hormone receptors. HR+: presence of either estrogen and/or progresterone hormone receptors. ODI3: oxygen desaturation index at 3%. T90: percentage of time spent with oxygen saturation <90%. SaO2: oxygen saturation. SDB: sleep-disordered breathing.
Data are expressed as median (first-third quartile).
(DOCX)
