# Supplementary material for: Association between sleep-disordered breathing and breast cancer aggressiveness
Source: PLoS One. 2018 Nov 21;13(11):e0207591. doi: 10.1371/journal.pone.0207591 (PMC6248981; doi:10.1371/journal.pone.0207591)
Supplement: S1 Appendix — (DOCX) [file pone.0207591.s001.docx]

**S1 Appendix: Methods**

Molecular subtypes. Based on the presence or absence of hormone receptors, the epidermal growth factor receptor 2 (HER2) status, and cell proliferation, the molecular subtypes were defined as follows: Luminal A subtype (estrogen or progesterone receptors positive, or both, HER2 negative, low tumor proliferation defined as a Ki67<20%); luminal B subtype (estrogen or progesterone receptors positive, or both, HER2 negative or positive, and high tumor proliferation defined as a Ki67>20%); HER2 subtype, (characterized by a high expression of the HER2 gene and both, estrogen and progesterone receptors negative); triple negative (HER2 negative and estrogen and progesterone receptors negative).[1–4]

Immunohistochemical assessment of the proportion of cells staining for the nuclear antigen Ki67 has been used to establish cell proliferation in breast cancer. There are consistent data on Ki67 as a strong prognostic marker in breast cancer. Ki67 values of 10% or less are generally considered low risk, and values between 20% and 29% are considered as a criterion for high proliferation.[1,4,5]

The Nottingham Histological Grade (NHG), is the most widely used histologic grading system of breast cancer.[6,7] NHG assesses tumor differentiation based on a subjective evaluation of morphological features, namely the percentage of tubule formation, the degree of nuclear pleomorphism and the mitotic count. Three grades are defined, grade I (well-differentiated), grade 2 (moderately differentiated), and grade 3 (poorly differentiated). There is a high correlation between histological grade and prognosis: both recurrence-free interval and overall survival are worse in patients with poorly differentiated tumors compared with those with well-differentiated tumors.[6]

Finally, the stage of the disease was assessed according to the American Joint Committee on Cancer (7th edition). Localized breast cancer was classified as stage I or II, locoregional disease as stage III, and distant metastases as stage IV.[8]

**Supplemental References**

1. Harbeck N, Gnant M. Breast cancer. Lancet. 2017;389: 1134–1150. doi:10.1016/S0140-6736(16)31891-8

2. Cheang MCU, Chia SK, Voduc D, Gao D, Leung S, Snider J, et al. Ki67 index, HER2 status, and prognosis of patients with luminal B breast cancer. J Natl Cancer Inst. 2009;101: 736–750. doi:10.1093/jnci/djp082

3. Eroles P, Bosch A, Pérez-Fidalgo JA, Lluch A. Molecular biology in breast cancer: intrinsic subtypes and signaling pathways. Cancer Treat Rev. 2012;38: 698–707. doi:10.1016/j.ctrv.2011.11.005

4. Coates AS, Winer EP, Goldhirsch A, Gelber RD, Gnant M, Piccart-Gebhart M, et al. Tailoring therapies--improving the management of early breast cancer: St Gallen International Expert Consensus on the Primary Therapy of Early Breast Cancer 2015. Ann Oncol. 2015;26: 1533–1546. doi:10.1093/annonc/mdv221

5. Dowsett M, Nielsen TO, A’Hern R, Bartlett J, Coombes RC, Cuzick J, et al. Assessment of Ki67 in breast cancer: recommendations from the International Ki67 in Breast Cancer working group. J Natl Cancer Inst. 2011;103: 1656–1664. doi:10.1093/jnci/djr393

6. Elston CW, Ellis IO. Pathological prognostic factors in breast cancer. I. The value of histological grade in breast cancer: experience from a large study with long-term follow-up. Histopathology. 1991;19: 403–410.

7. Rakha EA, El-Sayed ME, Lee AHS, Elston CW, Grainge MJ, Hodi Z, et al. Prognostic significance of Nottingham histologic grade in invasive breast carcinoma. J Clin Oncol. 2008;26: 3153–3158. doi:10.1200/JCO.2007.15.5986

8. Edge S, Bird D, Compton C, Fritz A, Greene F, Trotti A. AJCC cancer staging manual (7th ed). New York, NY: Springer; 2010.
