# Supplementary material for: Association between sleep-disordered breathing and breast cancer aggressiveness
Source: PLoS One. 2018 Nov 21;13(11):e0207591. doi: 10.1371/journal.pone.0207591 (PMC6248981; doi:10.1371/journal.pone.0207591)
Supplement: S1 Table — p-value was >0.05 for all comparisons between the group with Ki67>28% and Ki67<29% Data are expressed as mean (SD), median (first-third quartile), or number of patients (%). (DOCX) [file pone.0207591.s002.docx]

**S1 Table. Comparison of baseline general characteristics between the groups of women with more (Ki67>28%) and less aggressive (Ki67<29%) breast cancer.**

| **Variables** | **Ki67>28%**  **(n=41)** | **Ki67<29%**  **(n=42)** |
| --- | --- | --- |
| *General characteristics* |  |  |
| Age (years) | 47.9 (8.9) | 49.4 (8.8) |
| Body mass index (Kg/m2)  Body mass index≥30 Kg/m2 | 27.1 (4.6)  10 (24.3) | 27.3 (5.5)  14 (33.3) |
| Neck circumference (cm) | 34.4 (2.6) | 34.2 (3.5) |
| Waist-to-hip ratio | 0.85 (0.09) | 0.84 (0.08) |
| Post-menopausal status | 22 (53.6) | 20 (47.6) |
| Hormone replacement therapy | 2 (4.8) | 1 (2.3) |
| Familiar history of breast cancer | 10 (24.3) | 12 (28.5) |
| Active smoking | 9 (21.9) | 6 (14.2) |
| Night shift work >1 year | 6 (14.6) | 9 (21.4) |
| Number of hours of sleep in the last year | 7 (6-8) | 8 (7-8) |
| Epworth scale  Epworth scale>10 | 7 (4.2-8)  4 (9.7) | 4.5 (2-8)  6 (14.2) |

p-value was >0.05 for all comparisons between the group with Ki67>28% and Ki67<29%

Data are expressed as mean (SD), median (first-third quartile), or number of patients (%).
