# Supplementary material for: Association between sleep-disordered breathing and breast cancer aggressiveness
Source: PLoS One. 2018 Nov 21;13(11):e0207591. doi: 10.1371/journal.pone.0207591 (PMC6248981; doi:10.1371/journal.pone.0207591)
Supplement: S2 Table — SDB: sleep-disordered breathing. HR-: absence of estrogen and progresterone hormone receptors. HR+: presence of either estrogen and/or progresterone hormone receptors. ODI3: oxygen desaturation index at 3%. T90: percentage of time spent with oxygen saturation <90%. SaO2: oxygen saturation. SDB: sleep-disordered breathing. Data are expressed as median (first-third quartile). (DOCX) [file pone.0207591.s003.docx]

**S2 Table. Values for different oximetric parameters of SDB according to the categories of several aggressiveness markers of breast cancer.**

|  | ODI3% | p-value | T90 | p-value | Minimum SaO2 | p-value |
| --- | --- | --- | --- | --- | --- | --- |
| *Ki67*  Ki67>28%  Ki67<29% | 5.2 (2-9.5)  5.2 (2.3-10.9) | 0.78 | 0 (0-0.1)  0 (0-0.7) | 0.56 | 89 (85-91)  89 (84-91) | 0.93 |
|  |  |  |  |  |  |  |
| *Hormone receptors*  HR-  HR+ | 5.9 (1.2-14.7)  5.1 (2.4-10) | 0.92 | 0.1 (0-3.1)  0 (0-0.7) | 0.20 | 88 (83.5-91)  90 (85-91) | 0.64 |
|  |  |  |  |  |  |  |
| *Nottingham Histological Grade*  Grade 1  Grade 2  Grade 3 | 5.1 (2.7-10)  6.3 (1.5-10.1)  5 (2.5-12.1) | 0.94 | 0 (0-0.2)  0 (0-0.7)  0 (0-3.2) | 0.79 | 89 (83.5-90)  90 (85-91)  89 (85-91) | 0.78 |
|  |  |  |  |  |  |  |
| *Tumor stage*  Stage I  Stage II  Stage III-IV | 6.2 (1.7-12.5)  4.4 (2.4-10.39  5.1 (0.7-8.6) | 0.67 | 0 (0-0.8)  0 (0-1.3)  0 (0-0.4) | 0.82 | 89 (84-90)  89 (85-91.2)  90 (86.2-91) | 0.44 |
| *Molecular subtype*  Luminal A  Luminal B  HER2  Triple Negative | 5.2 (1.8-12.1)  4.8 (2.1-12.1)  6.3 (3.0-9.6)  3.7 (1.2-9.8) | 0.82 | 0 (0-0.1)  0 (0-0.7)  0 (0-0.7  0 (0-3.3) | 0.78 | 89 (84-90)  89 (83-91)  89 (84-91)  88 (85-91) | 0.99 |

SDB: sleep-disordered breathing. HR-: absence of estrogen and progresterone hormone receptors. HR+: presence of either estrogen and/or progresterone hormone receptors. ODI3: oxygen desaturation index at 3%. T90: percentage of time spent with oxygen saturation <90%. SaO2: oxygen saturation. SDB: sleep-disordered breathing.

Data are expressed as median (first-third quartile)
